# Epidemiology and Burden of Human Metapneumovirus Among Italian Adults in Outpatient and Inpatient Settings, 2014–2025

**DOI:** 10.1111/irv.70175

**Published:** 2025-10-20

**Authors:** Alexander Domnich, Donatella Panatto, Vincenzo Paolozzi, Matilde Ogliastro, Valentina Ricucci, Giancarlo Icardi, Andrea Orsi

**Affiliations:** ^1^ Hygiene Unit San Martino Polyclinic Hospital – IRCCS for Oncology and Neurosciences Genoa Italy; ^2^ Department of Health Sciences (DISSAL) University of Genoa Genoa Italy; ^3^ Interuniversity Research Centre on Influenza and Other Transmissible Infections (CIRI‐IT) Genoa Italy

**Keywords:** adults, disease burden, human metapneumovirus, older adults, surveillance

## Abstract

**Background:**

Human metapneumovirus (hMPV) has been increasingly recognized as a major contributor to respiratory infections in all age groups. Owing to its recent discovery, available data on the burden of hMPV in adults are still scant and heterogeneous. Here, we aimed to explore the epidemiology, symptomatic profile, and mortality related to hMPV among Italian adults.

**Methods:**

We performed an integrated analysis of several community‐based and hospital‐based studies conducted in Genoa (Italy) between 2014 and 2025. Adults aged ≥ 18 years prescribed with ≥ 1 molecular test for hMPV were eligible.

**Results:**

Of 21,580 and 2671 adults included in the hospital‐based and community‐based studies, 376 and 117, respectively, tested positive for hMPV. Seasonal (November to April) hMPV detection rate was 4.4% (95% CI: 3.6%–5.2%) in the community‐based and 2.4% (95% CI: 2.1%–2.6%) in the hospital‐based studies. Most detections occurred during the spring months. Each 1‐year increase in age was associated with a 1.1% increase in the odds of hMPV positivity (adjusted odds ratio [aOR] 1.011; 95% CI: 1.005–1.016). Clinical presentation of hMPV resembled that of the phylogenetically related respiratory syncytial virus. Among hMPV‐positive inpatients, 7.3% (95% CI: 4.3%–11.5%) died during their hospital encounter. In‐hospital mortality was associated with residency in long‐term care facilities (aOR 8.73; 95% CI: 2.63–29.15) and cancer (aOR 4.51; 95% CI: 1.50–14.35).

**Conclusions:**

hMPV is a common virological finding in outpatient and inpatient adults and is responsible for a measurable burden, especially among the most frail older adults.

## 1 Introduction

Following its discovery in 2001 [1], the human metapneumovirus (hMPV) has been increasingly recognized as an important cause of acute respiratory infections (ARIs) in both pediatric [2] and adult [3] populations. hMPV is an enveloped, negative‐strand RNA virus that, together with avian metapneumovirus, belongs to the genus *Metapneumovirus* within the family *Pneumoviridae*. hMPV shares several features with respiratory syncytial virus (RSV), which belongs to the same family *Pneumoviridae* [4]. Similar to RSV, hMPV expresses two major surface glycoproteins, F and G, where the former, a fusion protein, mediates viral entry and the latter contributes to viral attachment [5]. hMPV has two genetically and antigenically distinct lineages, A and B, each of which has diverged into several sub‐lineages [6].

Owing to its recent discovery and comparatively few commercially available molecular tests for its detection, the available estimates of hMPV incidence and prevalence vary substantially. A meta‐analysis by Lefebvre et al. [7] reported that 6.2% of hospitalized ARI patients tested positive for hMPV. However, this pooled proportion was associated with very high heterogeneity, and single‐study estimates ranged from 0% to 36.4%. Another meta‐analysis on adults aged ≥ 60 years living in high‐income countries estimated that 7.0% of all lower respiratory tract infections are due to hMPV [8]. Falsey [9] suggests that the incidence of symptomatic hMPV in the general adult population is typically below 5%. In any case, hMPV is ubiquitous, and by the age of 5 years, virtually all individuals have already been exposed to the virus [10].

It is thought that the range of symptoms associated with hMPV is similar to that of RSV, with cough, nasal congestion, and nonspecific systemic symptoms being most frequently reported [11, 12]. The overall hospitalization rate for hMPV in adults was estimated at 19 per 100,000, while in older adults aged ≥ 80 years, it reaches 130 per 100,000 [13]. Among hospitalized adults, positivity for hMPV is independently associated with severe respiratory disease requiring augmented oxygenation or ventilation and significantly prolonged hospital stay [14]. A multicountry study [15] based on 100 hMPV hospitalizations reported that 5%, 8%, and 2% of adults (≥ 18 years) required mechanical ventilation, were admitted to intensive care units, or died during the hospital stay, respectively. Notably, the hMPV in‐hospital mortality rate was similar to that of influenza (1.6%) and RSV (2.5%) [15]. An increasing understanding of a substantial hMPV burden in both children and adults has led to the development of several vaccine candidates (typically in combination with RSV), some of which have entered the clinical stage [16, 17, 18].

The available reviews [8, 9, 19] have underscored several knowledge gaps in our understanding of hMPV infection in adults. Furthermore, the available burden estimates appear heterogeneous, which is likely driven by a number of factors, including surveillance period, setting, case definition, age distribution, and other study design attributes. The objective of this study was to explore the epidemiology, clinical presentation, and mortality associated with hMPV infection among Italian adults.

## 2 Methods

### 2.1 Study Overview and Data Sources

This integrated analysis combines hospital‐based and community‐based surveillance studies conducted in Genoa (Italy).

For the hospital‐based study, we retrospectively collected all real‐time polymerase chain reaction (RT‐PCR) tests for the detection of hMPV and other respiratory pathogens performed at the San Martino Research Hospital between November 1, 2014, and April 30, 2024. The hospital is a 1200‐bed adult research tertiary care medical center that conducts routine respiratory surveillance activities also with the aim of contributing data to the national surveillance system RespiVirNet. RT‐PCR tests were performed mostly on hospitalized individuals, but also on outpatients, including emergency department (ED) visits with no subsequent hospitalization and specialist visits. All RT‐PCR tests were processed based on clinical requests. The overwhelming majority (> 99%) of samples were represented by nasopharyngeal (NP) or oropharyngeal swab specimens eluted in a transport medium. A few lower respiratory tract specimens (bronchoaspirates or bronchoalveolar lavage) could also be processed, but the specimen types could not be discerned. To be included in the study, subjects aged ≥ 18 years had to be prescribed ≥ 1 RT‐PCR for hMPV. We excluded those RT‐PCR tests for which no definite microbiological diagnosis could be inferred (e.g., inadequate sampling or inconclusive result). For patients with ≥ 1 positive hMPV test performed during the same season, only the first positive test was considered. RT‐PCR records of hMPV‐positive individuals were linked to the available patient files, and relevant medical history and clinical data (see below) were collected. However, data linkage could be performed only for hospitalized individuals whose patient files were digitalized.

Community‐based studies were conducted during two pre‐COVID‐19 pandemic seasons 2018/2019 (November 2018 to April 2019) and 2019/2020 (November 2019 to mid‐March 2020; terminated earlier due to COVID‐19) and two post‐pandemic seasons 2023/2024 (mid‐October 2023 to April 2024) and 2024/2025 (mid‐October 2024 to April 2025). The two pre‐pandemic studies aimed to assess influenza vaccine effectiveness (through test‐negative design) and were conducted within the DRIVE (Development of Robust and Innovative Vaccine Effectiveness) project. A detailed description of the project can be accessed elsewhere [20, 21]. Briefly, sentinel general practitioners (GPs) enrolled community‐dwelling adults (≥ 18 years) seeking care for influenza‐like illness (ILI). ILI was defined according to the European criteria [22], as follows: abrupt onset of ≥ 1 systemic (fever/feverishness, malaise, headache, myalgia) and ≥ 1 respiratory (cough, sore throat, shortness of breath) symptom. Subjects with symptom onset > 7 days before the GP visit and residents of long‐term care facilities (LTCFs) were excluded. During the visit, GPs collected demographic and clinical data and all eligible subjects underwent a NP swab that was tested for respiratory pathogens, including hMPV.

The post‐pandemic study was conducted within the RESPIRA‐50 project on RSV epidemiology in primary care. Further details on the study can be found elsewhere [23, 24]. In brief, 24 (season 2023/2024) and 36 (season 2024/2025) sentinel GPs were randomized 1:1 into two groups. The first enrolled adults aged ≥ 50 years seeking care for ARI defined according to the European criteria (sudden onset of ≥ 1 respiratory symptom [cough, sore throat, shortness of breath, coryza] and GP's judgment of an underlying infection) [22]. The second group enrolled ≥ 50‐year‐olds who sought care for ILI defined as per the European case definition. A total of 93% of outpatients enrolled in the ARI group also met the ILI case definition, while all ILI patients also met the ARI criteria. LTCF residents and subjects with a swab delay > 7 days were excluded. For all individuals, relevant sociodemographic and clinical data were collected, and a NP swab was taken.

### 2.2 RT‐PCR

RT‐PCR for the detection of hMPV and other respiratory pathogens (influenza A and B; RSV A and B; adenovirus; enterovirus; parainfluenza viruses 1–4; bocaviruses 1–4; coronaviruses 229E, NL63, OC43; rhinovirus; *Streptococcus pneumoniae* ; *Bordetella parapertussis* ; *Bordetella pertussis* ; *Chlamydophila pneumoniae* ; *Haemophilus influenzae* ; *Legionella pneumophila* ; *Mycoplasma pneumoniae* ) was performed using the Allplex Respiratory Panels 1–4 (Seegene Inc.; Seoul, Korea) according to the manufacturer's instructions and as previously described [24, 25]. This RT‐PCR assay was used in all outpatient and inpatient studies and was performed at the same laboratory. Samples with cycle threshold values < 40 were deemed positive.

### 2.3 Study Variables

For both hospital‐based and community‐based studies, the primary outcome was hMPV prevalence, defined as a proportion of hMPV‐positive individuals to the total number of subjects tested. This endpoint was described by age groups (18–49, 50–64, 65–74, and ≥ 75 years), calendar month, and co‐detection pattern (single hMPV detections vs. hMPV co‐detected with other pathogens). Notably, prevalence rates estimated in the hospital‐based studies should not be directly compared with those estimated in the community‐based studies because hospital‐based surveillance activities were year‐round, while the community‐based studies were limited to a typical respiratory season (mid‐October/November to April). To allow for a more consistent comparison, we also restricted the hospital‐based dataset to a comparable period (November to April).

For the community‐based studies, the prevalence of different symptoms among hMPV‐positive individuals was of interest. Specifically, for the RESPIRA‐50 study, a comprehensive set of systemic (feverishness, measured fever, shivering, headache, myalgia, arthralgia, malaise, decreased appetite, nausea, diarrhea) and respiratory (cough, cough with sputum, dyspnea, tachypnea, rhonchi, wheezing, need for oxygen supply, low/decreased saturation, sore throat, coryza, altered smell, altered taste) signs and symptoms was gathered. The list of symptoms collected during the DRIVE studies was limited to those qualifying for the ILI case definition. Finally, for the hospital‐based study, the in‐hospital case‐fatality rate was estimated. This latter outcome was defined as the proportion of hMPV‐positive individuals who died from any cause during the current hospital encounter to the total number of hospitalized individuals who tested positive for hMPV.

Independent variables considered are sex, age, residency in LTCFs, and the presence of comorbidities (diabetes, cardiovascular, respiratory, hepatic, renal, neurodegenerative, rheumatic diseases, cancer, and other immunosuppressive conditions).

### 2.4 Statistical Analysis

hMPV prevalence and other categorical variables were expressed as percentages with Clopper‐Pearson's exact 95% confidence intervals (CIs), while continuous predictors were described by means with standard deviations (SDs). Correlation between monthly prevalence and the number of tests was assessed via Spearman's rho. Effect sizes were reported as odds ratios (ORs). Specifically, the association between hMPV positivity and age was assessed via a multivariable logistic model with adjustment for sex, setting (hospital vs. community), and year. Owing to the paucity of hMPV‐related deaths, Firth's penalized logistic regression was applied to identify significant predictors of in‐hospital mortality, which were selected by minimizing the Akaike information criterion (AIC).

Data analysis was performed using the R software v. 4.3.3 (R Foundation for Statistical Computing; Vienna, Austria).

## 3 Results

### 3.1 Epidemiology of hMPV

For the hospital‐based study, we identified 21,580 hospitalized adults who underwent ≥ 1 RT‐PCR test for the detection of hMPV between November 2014 and April 2024. In the community‐based studies, 2671 adults were prospectively enrolled during four winter seasons and tested for hMPV.

In the hospital‐based study, 376 (1.7%; 95% CI: 1.6%–1.9%) subjects tested positive for hMPV year‐round. If restricted to the period between November and April, hMPV prevalence rose to 2.4% (329/13,948; 95% CI: 2.1%–2.6%). In the community‐based studies, 117 hMPV detections were registered, and the overall prevalence was 4.4% (95% CI: 3.6%–5.2%). hMPV prevalence increased with age, reaching 2.1% and 5.7% in adults aged ≥ 75 years enrolled in the hospital‐based and community‐based studies, respectively (Table 1). After adjustment for sex, setting, and year, each 1‐year increase in age was associated (*p* < 0.001) with a 1.1% increase in the odds (OR 1.011; 95% CI: 1.005–1.016) of testing positive for hMPV.

**TABLE 1:** Prevalence of human metapneumovirus (hMPV) detections in hospital‐based (*n* = 21,580) and community‐based (*n* = 2671) studies, by age group.

Age, years	Hospital‐based study (year‐round)		Hospital‐based study (November to April)		Community‐based study (mid‐October/November to April)	
	% (*n*/Total)	95% CI	% (*n*/Total)	95% CI	% (*n*/Total)	95% CI
18–49	0.8 (30/3599)	0.6–1.2	1.3 (27/2154)	0.8–1.8	2.5 (14/567)	1.4–4.1
50–64	1.5 (68/4544)	1.2–1.9	2.0 (55/2810)	1.5–2.5	4.0 (39/963)	2.9–5.5
65–74	1.9 (86/4461)	1.5–2.4	2.6 (76/2901)	2.1–3.3	5.5 (31/559)	3.8–7.8
≥ 75	2.1 (192/8976)	1.8–2.5	2.8 (171/6083)	2.4–3.3	5.7 (33/582)	3.9–7.9

Abbreviation: CI, confidence interval.

**FIGURE 1:**
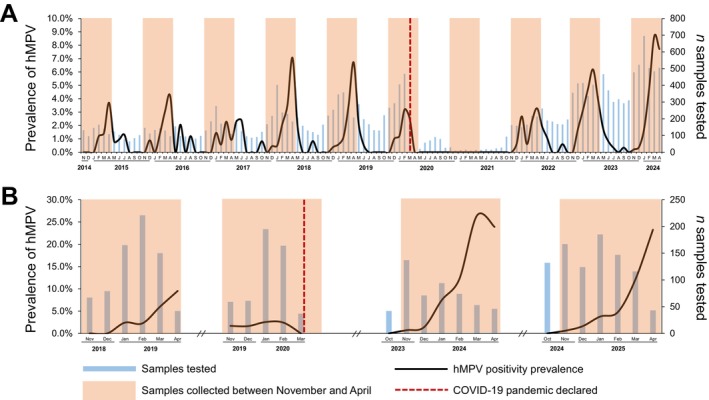
Monthly distribution of real‐time polymerase chain reaction tests and human metapneumovirus (hMPV) positivity prevalence in the hospital‐based (A) and community‐based (B) studies.

As shown in Figure 1, hMPV rates progressively increased from autumn to spring months, with peaks more commonly observed in April. Indeed, the average April detection rates were 5.4% (95% CI: 4.4%–6.5%) and 19.1% (95% CI: 12.8%–26.9%) in the hospital‐based and community‐based studies, respectively. Notably, some out‐of‐season hMPV activity was also recorded in the hospital‐based study: The average prevalence between May and September was 0.7% (95% CI: 0.5%–1.0%) (Figure S1). The highest peak prevalence was observed during the pre‐COVID‐19 seasons 2017/2018 and 2018/2019 and the post‐pandemic season 2023/2024. Conversely, no detections occurred between April 2020 and November 2021 (Figure 1). In the hospital‐based study, monthly hMPV detection rates positively correlated (rho = 0.54, *p* < 0.001) with the monthly number of tests performed.

### 3.2 Co‐Detections in Patients Tested Positive for hMPV

At least one other pathogen was detected in 18.9% (71/376) of hMPV‐positive patients in the hospital‐based study and 41.9% (49/117) of outpatients in the community‐based studies. Most co‐detections were due to bacteria (hospital‐based study: 17.0%; community‐based study: 30.8%), especially *Haemophilus influenzae* (hospital‐based study: 9.8%; community‐based study: 21.4%) and *Streptococcus pneumoniae* (hospital‐based study: 8.9%; community‐based study: 11.1%). Viral co‐detections were less common in both hospital‐based (6.4%) and community‐based (15.4%) studies. The most common viral co‐detections were rhinovirus observed in 2.9% of inpatients and 4.3% of outpatients, while co‐detections with influenza, RSV, and SARS‐CoV‐2 were uncommon (Table S1).

### 3.3 Characteristics of Patients Tested Positive for hMPV

Of 376 hMPV‐positive subjects identified in the hospital‐based study, 306 (81.4%) were from hospitalized adults, while the remaining 19 (5.1%) were ED referrals without subsequent hospitalization, and 51 (13.6%) were from specialist visits without overnight stays. Of 306 inpatient stays, patient files of 232 (75.8%) individuals could be retrieved. As shown in Table 2, their mean age was 76.0 years, and females slightly prevailed (53.4%). A total of 9.5% of subjects were LTCF residents. Most (91.8%) individuals had ≥ 1 comorbidity, of which cardiovascular (70.3%) and respiratory (28.4%) diseases and cancer (25.9%) were the most prevalent.

**TABLE 2:** Characteristics of adults who tested positive for human metapneumovirus (hMPV), by setting.

Characteristic	Level	Hospital‐based study (*n* = 232), *n* (%)	Community‐based studies (*n* = 117), *n* (%)
Sex	Male	108 (46.6)	52 (44.4)
	Female	124 (53.4)	65 (55.6)
Age, years	Mean (SD)	76.0 (13.4)	64.9 (15.6)
	18–49	9 (3.9)	14 (12.0)
	50–64	36 (15.5)	39 (33.3)
	65–74	50 (21.6)	31 (26.5)
	≥ 75	137 (59.1)	33 (28.2)
Underlying health conditions	≥ 1	207 (89.2)	63 (53.8)
	Cardiovascular	163 (70.3)	49 (41.9)
	Respiratory	66 (28.4)	19 (16.2)
	Diabetes	41 (17.7)	9 (7.7)
	Hepatic	13 (5.6)	2 (1.7)
	Renal	35 (15.1)	11 (9.4)
	Neurodegenerative	19 (8.2)	2 (1.7)
	Cancer	60 (25.9)	11 (9.4)
	Immunosuppression	13 (5.6)	2 (1.7)
LTCF resident	Yes	22 (9.5)	0 (0)^a^
	No	210 (90.5)	117 (100)

Abbreviations: IQR, interquartile range; LCTF, long‐term care facility; SD, standard deviation.

^a^ Residency in long‐term care facility (LTCF) was the exclusion criterion.

hMPV‐positive adults (*n* = 117) enrolled in the community‐based studies were much younger (mean age 64.9 years) and consequently had a lower prevalence (53.8%) of comorbidities than hospitalized adults (Table 2).

### 3.4 Symptomatic Profile of the hMPV Infection Among Community‐Dwelling Adults

**FIGURE 2:**
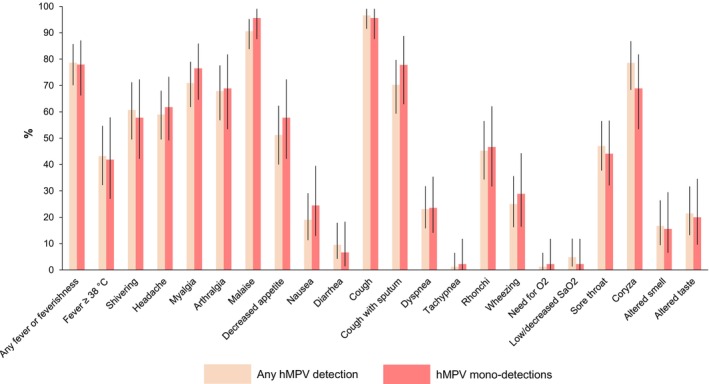
Frequency of systemic and respiratory signs and symptoms reported by outpatient adults with any positivity to human metapneumovirus (hMPV) or with single hMPV detections.

In hMPV‐positive outpatients, malaise (90.6%; 106/117) was the most common systemic symptom. Any fever or feverishness was recorded in 78.6% (92/117) of subjects. However, fever ≥ 38°C was relatively uncommon (43.2%; 35/81). The most common respiratory symptoms were cough (96.6%; 113/117) and coryza (78.6%; 66/84). Symptom prevalence was similar when only hMPV mono‐detections were considered (Figure 2; Table S2).

**FIGURE 3:**
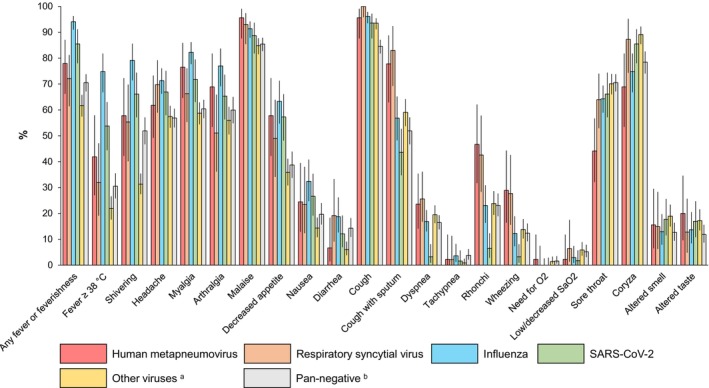
Comparison of the symptomatic profiles across different single viral etiologies. ^a^Positive for adenovirus, human rhinovirus, enterovirus, bocaviruses, parainfluenza viruses, or seasonal coronaviruses. ^b^Negative for all viruses and bacteria tested.

Among mono‐detections caused by single viruses, hMPV and RSV showed the most similar symptomatic profile. For instance, the prevalence of fever ≥ 38°C was the highest among influenza mono‐detections (74.8%; 104/139), which was significantly higher than among both hMPV (41.9%; 18/43) and RSV (31.9%; 15/47) mono‐detections. Compared with RSV, hMPV mono‐detections showed a lower frequency of sore throat (44.1% vs. 68.9%) and coryza (68.9% vs. 87.2%), but the 95% CIs overlapped (Figure 3; Table S3).

### 3.5 In‐Hospital Mortality Associated With hMPV

Of 232 hMPV‐positive hospitalized individuals with available patient files, 17 (7.3%; 95% CI: 4.3%–11.5%) died during their hospital stay. Their mean age was 80.1 (SD 11.2; range 56–100) years, and 52.9% (9/17) were females. All subjects had ≥ 1 comorbidity with cardiovascular (76.5%; 13/17), respiratory (41.2%; 7/17) diseases, and cancer (47.1%; 8/17) being the most prevalent. In a multivariable model, residency in LTCFs (OR 8.73; 95% CI: 2.63–29.15; *p* < 0.001) and cancer (OR 4.51; 95% CI: 1.50–14.35; *p* = 0.008) were associated with death. The presence of respiratory disease increased the odds of death by 2.29, but the 95% CI included the null (0.76–6.76; *p* = 0.14) (Table S4).

## 4 Discussion

In recent years, adult hMPV infection has been increasingly recognized as a public health concern [26], contributing significantly to the burden of respiratory illness alongside influenza and RSV [27], and, more recently, SARS‐CoV‐2. To address this unmet need, several vaccine candidates are in clinical development [16, 17, 18]. Available systematic reviews [7, 8, 28, 29] have underlined several research gaps, including a scarcity of data on older adults, community settings, and the limited geographic coverage of the primary studies. This integrated analysis of adult inpatient and outpatient studies conducted during the last decade aimed to fill some research gaps on the epidemiology and burden of hMPV in Italy and Europe.

The overall detection of hMPV was 1.7% in the hospital‐based study (2.4% within the respiratory season) and 4.4% in the community‐based studies. In a meta‐analysis of 21 studies conducted in 10 high‐income countries [8], the pooled detection rate of hMPV among adults aged ≥ 60 years with lower respiratory infections was estimated at 7.0% (95% CI: 5.4%–9.1%). However, the heterogeneity was substantial (*I* ^2^ = 94%) with single‐study proportions ranging ninefold, from 2.3% to 21.4%. The main drivers of this heterogeneity were attributed to the differences in study designs, such as country, setting, season, and case definitions. Notably, pooling of two Italian studies showed the lowest hMPV prevalence of 2.8% (95% CI: 1.9%–4.1%) [8]. In a very recent Italian study conducted in nine regions between September 2022 and August 2024 [30], which was not covered by the above‐mentioned meta‐analysis, the all‐age hMPV prevalence among individuals with ARI/ILI was 3.4%. Prevalence was lower (2.6%) among adults aged ≥ 50 years. Similar to our findings, hMPV was more common among outpatients (5.1%) than among inpatients (3.5%) [30].

Like RSV, previous observations suggest [4, 8] that the burden of hMPV follows a U‐shape age distribution pattern, in which young children and the oldest old are at the highest risk of disease and severe outcomes. Our findings are in line with this: hMPV prevalence was the lowest in young adults and the highest in those aged ≥ 75 years independently of setting. In Shanghai (2014–2023) [31], positivity rates in adults aged ≥ 65 years were higher than those in working‐aged adults in both outpatient (2.31% vs. 1.36%) and inpatient (2.16% vs. 1.91%) settings. In France (2012–2018) [32], being > 65 years was independently associated with an increased risk (OR 3.3; 95% CI: 1.9–6.1) of hMPV detection. Interestingly, in that French hospital‐based study, the median age of hMPV‐positive individuals was higher than that positive for influenza (78 vs. 69 years; *p* < 0.001) and RSV (78 vs. 74 years; *p* = 0.06) [32]. Within older adults, a model [8] predicted a steady increase in the incidence of hMPV from 50 to 59 to ≥ 75 years. This age‐related pattern should undoubtedly be considered for future prevention strategies.

Although circulation of hMPV overlaps with that of other respiratory viruses, its seasonality pattern is distinct. In our study, peaks were typically observed between March and May with an average 10‐year peak prevalence maximized in April. A Korean nationwide five‐season time‐series analysis [33] defined hMPV as the only “spring virus,” as it always peaked between March and May. In that study, hMPV peaked later than influenza, RSV, and coronaviruses (“winter viruses”) and slightly before bocaviruses and parainfluenza viruses (“spring/summer viruses”) [33]. In a 10‐season US study [34], hMPV and RSV epidemics (defined as ≥ 3% positive tests in the last of ≥ 2 consecutive weeks) overlapped for a median of 13.5 (range 11–19) weeks before the COVID‐19 pandemic and for a much shorter period (median 3 weeks; range 1–10 weeks) during and after the pandemic. hMPV peak circulation always followed that of RSV by a median of 11.5 weeks before the pandemic and 19 weeks during and after the pandemic [34].

We then showed that an increasing number of samples processed was temporally associated with an increased prevalence of hMPV detections, which was particularly pronounced in the post‐COVID‐19 period. An analogous post‐pandemic recrudescence was observed for other respiratory viruses like RSV, and several underlying mechanisms have been proposed, including the so‐called “immunity debt” theory, interaction between SARS‐CoV‐2 and other viruses, and SARS‐CoV‐2‐induced immune dysregulation [35]. Alternatively, this increase may also be linked to changes in care seeking or testing behaviors. Relative to the pre‐pandemic phase, Petros et al. [36] reported a 2.4‐fold increase in the 2021–2023 RSV pediatric patient volume, which was associated with an 18.9‐fold increase in RSV test volume. The same trend was observed in our study. We believe that the increased usage of RT‐PCR multiplex tests is driven by an awareness of both multiplex RT‐PCR test availability and the burden of different respiratory pathogens. Consequently, a greater pool of subjects with less severe respiratory infections has been tested in the post‐pandemic phase, which, in turn, determined higher detection rates.

In this study, viral co‐detections among hMPV‐positive adults were much less common than bacterial co‐detections with *Streptococcus pneumoniae* and *Haemophilus influenzae* . Specifically, co‐detections with influenza, RSV, and SARS‐CoV‐2 were rare, which is likely driven by the above‐discussed temporal shift in virus circulation. Most viral co‐detections were due to rhinovirus that circulates year‐round with some recrudescence during school periods [33]. In a French inpatient study [32], the prevalence of viral co‐detections was 10%, while a US community‐based study [37] reported a prevalence of 5.2%. Regarding bacterial co‐detections, their frequency reflects a high prevalence of *Streptococcus pneumoniae* [38] and *Haemophilus influenzae* [39] carriage among Italian adults. It should be stressed that the overall co‐detection prevalence intrinsically depends on the number of targets included in RT‐PCR kits used: The greater the number of targets, the higher the co‐detection prevalence is expected. Therefore, between‐study comparisons should be performed contextually.

Clinical presentation of hMPV‐related ARIs largely overlaps with those caused by other viruses; the former is more similar to RSV, which reflects the phylogenetic relatedness of hMPV and RSV. Malaise, cough, and coryza were the most common symptoms recorded in more than three‐quarters of ARI patients. This finding is consistent with data from a US study [37], in which fatigue (94.1%) and nasal congestion (83.7%) were the two most frequently reported symptoms among adults with ARI. Importantly, frequency of individual symptoms is study‐specific, as it depends on the pre‐specified set of symptoms collected. Furthermore, the symptom prevalence is likely higher in prospective studies, where symptoms are actively collected by a standardized tool. Conversely, retrospective studies based on patient files may be less accurate, as healthcare providers may register only the most prominent signs and symptoms. In proof, a systematic review by Sobanjo‐Ter Meulen et al. [29] reported the prevalence of fatigue ranging from 16.7% to 100%. In any case, the absence of clearly distinctive symptoms makes it challenging to differentiate hMPV from RSV and common viruses.

In a nutshell, hMPV is a common cause of acute upper and lower respiratory infections among community‐dwelling and hospitalized adults. As such, clinicians should consider including hMPV in the diagnostic pathway of ARIs, especially during the spring months and for patients who test negative for influenza, RSV, and SARS‐CoV‐2. Although there is currently no etiological treatment for hMPV [4], the availability of the virological diagnosis may reduce inappropriate antibiotic prescriptions. Commercially available laboratory‐based multiplex RT‐PCR kits also targeting hMPV have become increasingly common [40] and most large hospitals and medical centers have already adopted these assays [30]. On the other hand, the currently available near‐patient or point‐of‐care rapid molecular assays, which are often used in EDs, LTCFs, or other settings with no laboratory facilities, typically cover only SARS‐CoV‐2, influenza A/B, and RSV [41]. If not re‐tested using an alternative method, hMPV could be underdiagnosed.

A total of 7.3% of hMPV‐positive inpatients died during their hospital encounter. The most recent meta‐analysis [29] estimated the pooled case‐fatality rate in high‐risk adults (≥ 18 years) at 9.3% (95% CI: 4.6%–18.0%), which is close to our data. Analogously, the in‐hospital case‐fatality rates among older adults (≥ 50 years) ranged from 0% to 8.7%. Notably, those authors noted that most primary studies were based on an extremely low number of the total hMPV cases (< 20) [29], and consequently a higher probability of zero events. In this study, the in‐hospital mortality was associated with residency in LTCFs and the presence of cancer. This is in line with the pooled rates established by the above‐mentioned meta‐analysis [29]: Case‐fatality rates were 30.0% (95% CI: 16.4%–48.3%) among institutionalized elderly and 22.8% (95% CI: 13.4%–36.0%) among hematological malignancy patients. The risk of severe hMPV‐related outcomes is therefore skewed to the frailest individuals.

Together with its strengths, such as a long surveillance period, a comparatively large number of subjects tested for hMPV, and consistency of laboratory methods, we have to acknowledge some important study limitations. First, no clinical case definition was used in the hospital‐based study, but RT‐PCR testing was performed on clinical request. This may lead to selection bias. Second, data linkage between the laboratory findings and patient files in the hospital‐based study could be performed only on 75.8% of inpatients because several patient files related to some early (before 2017) episodes were not digitalized. Third, community‐based studies were not designed to evaluate the natural history of hMPV, and therefore, only clinical data related to the initial GP visit were available. Considering that several vaccine candidates containing the hMPV antigen are in clinical development, prospectively collected and country‐specific data on the complication, hospitalization, and mortality rates and associated resource consumption are needed to inform future health technology assessment studies. Fourth, despite the application of robust statistical techniques, the analysis on the correlates of hMPV mortality was likely underpowered because of a limited number of events. In this regard, some predictors were associated with comparatively large effect sizes but the 95% CIs were large and included the null. Finally, the study was conducted in a limited geographic area, which may impact the representativeness of our data.

In conclusion, hMPV is a common cause of adult ARIs and should be considered in the diagnostic pathways, especially during the spring months. Severe hMPV‐related outcomes are common among the elderly, especially if frail and immunocompromised. These population groups should be considered the primary targets of the eventual vaccination policies. Despite the fact that several countries, such as Italy, have recently incorporated detection of hMPV into the existing influenza surveillance platforms, awareness of hMPV is likely insufficient even in the medical community and should be improved.

## Author Contributions

**Alexander Domnich:** conceptualization, investigation, funding acquisition, writing – original draft, methodology, formal analysis. **Donatella Panatto:** investigation, funding acquisition, writing – review and editing, project administration. **Vincenzo Paolozzi:** data curation, investigation, writing – review and editing, validation. **Matilde Ogliastro:** investigation, writing – review and editing, project administration. **Valentina Ricucci:** investigation, writing – review and editing. **Giancarlo Icardi:** writing – review and editing, resources, supervision, funding acquisition. **Andrea Orsi:** conceptualization, investigation, formal analysis, methodology, writing – review and editing.

## Ethics Statement

This work was conducted in accordance with the Helsinki Declaration of 1964 and its later amendments. Prospective community–based studies were approved by the Liguria Region Ethics Committee (protocols 315/2018, 246/2019, and 397/2023), and written informed consent was obtained from all patients. Additionally, the hospital‐based study was conducted within the routine respiratory surveillance.

## Conflicts of Interest

a.d. provided consultancies and/or received speaker fees from CSL Seqirus, GSK, Sanofi, and SD Biosensor. D.P. provided consultancies for Pfizer and CSL Seqirus and received grants for conducting observational studies from Sanofi, Pfizer, GSK, and Viatris. G.I. provided consultancies and/or received grants for conducting experimental and/or observational studies for GSK, Sanofi, MSD, CSL Seqirus, and Pfizer. A.O. provided consultancies and/or received speaker fees from CSL Seqirus, Moderna, Novavax, and SD Biosensor. V.P., M.O., and V.R. declare no conflicts of interest.

## Peer Review

The peer review history for this article is available at https://www.webofscience.com/api/gateway/wos/peer‐review/10.1111/irv.70175.

## Supporting information


**Figure S1:** Average monthly human metapneumovirus (hMPV) positivity prevalence in the hospital‐based (*n* = 21,580) and community‐based studies (*n* = 2671).
**Table S1:** Frequency of viral and bacterial co‐detections in adults who tested positive for human metapneumovirus (hMPV), by setting.
**Table S2:** Frequency of systemic and respiratory signs and symptoms reported by outpatient adults with any positivity to human metapneumovirus (hMPV) or with single hMPV detections.
**Table S3:** Comparison of the symptomatic profiles across different single viral etiologies.
**Table S4:** Association between in‐hospital mortality among hospitalized adults who tested positive for human metapneumovirus (*n* = 232).

## Data Availability

All data generated and analyzed during this study are included in this article and its Supporting Information. Raw data generated during the current study are not publicly available due to CIRI‐IT internal policy and local ethical restrictions. Subject to certain criteria, conditions, and exceptions, and upon a reasonable request from qualified researchers, CIRI‐IT may provide access to the data.
