# Supplementary material for: Epidemiology and Burden of Human Metapneumovirus Among Italian Adults in Outpatient and Inpatient Settings, 2014–2025
Source: Influenza Other Respir Viruses. 2025 Oct 20;19(10):e70175. doi: 10.1111/irv.70175 (PMC12537271; doi:10.1111/irv.70175)
Supplement: Supplementary file 1 — Figure S1: Average monthly human metapneumovirus (hMPV) positivity prevalence in the hospital‐based (n = 21,580) and community‐based studies (n = 2671). Table S1: Frequency of viral and bacterial co‐detections in adults who tested positive for human metapneumovirus (hMPV), by setting. Table S2: Frequency of systemic and respiratory signs and symptoms reported by outpatient adults with any positivity to human metapneumovirus (hMPV) or with single hMPV detections. Table S3: Comparison of the symptomatic profiles across different single viral etiologies. Table S4: Association between in‐hospital mortality among hospitalized adults who tested positive for human metapneumovirus (n = 232). [file IRV-19-e70175-s001.docx]

**FIGURE S1** Average monthly human metapneumovirus (hMPV) positivity prevalence in the hospital-based (*n* = 21,580) and community-based studies (*n* = 2,671).


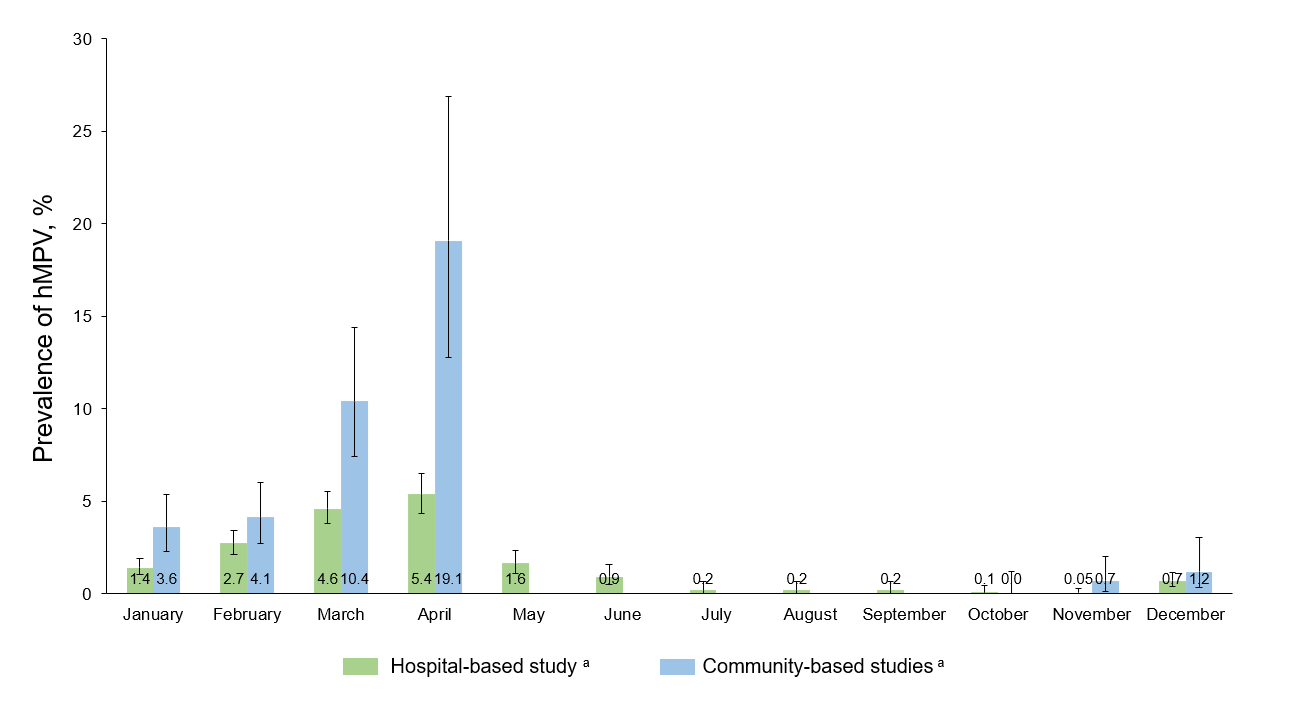


^a^ Surveillance activity was conducted year-round in the hospital-based study and between October and April in the community-based studies.

**TABLE S1** Frequency of viral and bacterial co-detections in adults who tested positive for human metapneumovirus (hMPV), by setting.

| **Pathogen** | | **% (*n*/Total) ^a^** | |
| --- | --- | --- | --- |
|  |  | **Hospital-based study** | **Community-based studies** |
| Viral co-detections | ≥ 1 virus | 6.4 (24/376) | 15.4 (18/117) |
|  | Influenza A | 0.3 (1/376) | 3.4 (4/117) |
|  | Influenza B | 0.3 (1/376) | 0 (0/117) |
|  | Human rhinovirus | 2.9 (11/376) | 4.3 (5/117) |
|  | Respiratory syncytial virus A | 0 (0/376) | 0.9 (1/117) |
|  | Respiratory syncytial virus B | 0.8 (3/376) | 1.7 (2/117) |
|  | Seasonal coronaviruses | 1.1 (4/376) | 2.6 (3/117) |
|  | SARS-CoV-2 | 0.3 (1/376) | 0 (0/117) |
|  | Parainfluenza virus 1 | 0 (0/376) | 0 (0/117) |
|  | Parainfluenza virus 2 | 0 (0/376) | 0 (0/117) |
|  | Parainfluenza virus 3 | 0.5 (2/376) | 1.7 (2/117) |
|  | Parainfluenza virus 4 | 0 (0/376) | 0 (0/117) |
|  | Adenovirus | 0.3 (1/376) | 1.7 (2/117) |
|  | Bocavirus | 0.3 (1/376) | 0 (0/117) |
|  | Enterovirus | 0 (0/376) | 0 (0/117) |
| Bacterial co-detections | ≥ 1 bacterium | 17.0 (52/305) | 30.8 (36/117) |
|  | *Streptococcus pneumoniae* | 8.9 (27/305) | 11.1 (13/117) |
|  | *Bordetella pertussis* | 0 (0/305) | 0 (0/117) |
|  | *Chlamydophila pneumoniae* | 0 (0/305) | 0 (0/117) |
|  | *Hemophilus influenzae* | 9.8 (30/305) | 21.4 (25/117) |
|  | *Legionella pneumophila* | 0 (0/305) | 0 (0/117) |
|  | *Mycoplasma pneumoniae* | 0 (0/305) | 0.9 (1/117) |

^a^ Numbers do not sum because of the presence of triple and quadruple detections.

**TABLE S2** Frequency of systemic and respiratory signs and symptoms reported by outpatient adults with any positivity to human metapneumovirus (hMPV) or with single hMPV detections.

| **Sign or symptom** | **Any hMPV detection** | | **hMPV mono-detection** | |
| --- | --- | --- | --- | --- |
|  | **% (*n*/Total)** | **95% CI** | **% (*n*/Total)** | **95% CI** |
| Any fever or feverishness | 78.6 (92/117) | 70.1–85.7 | 77.9 (53/68) | 66.2–87.1 |
| Fever ≥ 38 °C | 43.2 (35/81) | 32.2–54.7 | 41.9 (18/43) | 27.0–57.9 |
| Shivering | 60.7 (51/84) | 49.5–71.2 | 57.8 (26/45) | 42.2–72.3 |
| Headache | 59.0 (69/117) | 49.5–68.0 | 61.8 (42/68) | 49.2–73.3 |
| Myalgia | 70.9 (83/117) | 61.8–79.0 | 76.5 (52/68) | 64.6–85.9 |
| Arthralgia | 67.9 (57/84) | 56.8–77.6 | 68.9 (31/45) | 53.4–81.8 |
| Malaise | 90.6 (106/117) | 83.8–95.2 | 95.6 (65/68) | 87.6–99.1 |
| Decreased appetite | 51.2 (43/84) | 40.0–62.3 | 57.8 (26/45) | 42.2–72.3 |
| Nausea | 19.0 (16/84) | 11.3–29.1 | 24.4 (11/45) | 12.9–39.5 |
| Diarrhea | 9.5 (8/84) | 4.2–17.9 | 6.7 (3/45) | 1.4–18.3 |
| Cough | 96.6 (113/117) | 91.5–99.1 | 95.6 (65/68) | 87.6–99.1 |
| Cough with sputum | 70.2 (59/84) | 59.3–79.7 | 77.8 (35/45) | 62.9–88.8 |
| Dyspnea | 23.1 (27/117) | 15.8–31.8 | 23.5 (16/68) | 14.1–35.4 |
| Tachypnea | 1.2 (1/84) | 0.0–6.5 | 2.2 (1/45) | 0.1–11.8 |
| Rhonchi | 45.2 (38/84) | 34.3–56.5 | 46.7 (21/45) | 31.7–62.1 |
| Wheezing | 25.0 (21/84) | 16.2–35.6 | 28.9 (13/45) | 16.4–44.3 |
| Need for O_2_ | 1.2 (1/84) | 0.0–6.5 | 2.2 (1/45) | 0.1–11.8 |
| Decreased SaO_2_ | 4.8 (4/83) | 1.3–11.9 | 2.2 (1/45) | 0.1–11.8 |
| Sore throat | 47.0 (55/117) | 37.7–56.5 | 44.1 (30/68) | 32.1–56.7 |
| Coryza | 78.6 (66/84) | 68.3–86.8 | 68.9 (31/45) | 53.4–81.8 |
| Altered smell | 16.7 (14/84) | 9.4–26.4 | 15.6 (7/45) | 6.5–29.5 |
| Altered taste | 21.4 (18/84) | 13.2–31.7 | 20.0 (9/45) | 9.6–34.6 |

CI = confidence interval.

**TABLE S3** Comparison of the symptomatic profiles across different single viral etiologies.

| **Sign or symptom** | **% (*n*/Total)** | | | | | |
| --- | --- | --- | --- | --- | --- | --- |
|  | **hMPV** | **RSV** | **Influenza** | **SARS-CoV-2** | **Other viruses ^a^** | **Pan-negative ^b^** |
| Any fever or feverishness | 77.9 (53/68) | 72.1  (62/86) | 94.1 (318/338) | 85.5  (106/124) | 61.7  (333/540) | 70.5  (524/743) |
| Fever ≥ 38 °C | 41.9 (18/43) | 31.9  (15/47) | 74.8 (104/139) | 53.8  (64/119) | 21.9  (75/343) | 30.5  (111/364) |
| Shivering | 57.8 (26/45) | 55.3  (26/47) | 79.1 (110/139) | 66.1  (82/124) | 31.3  (169/540) | 51.9  (193/372) |
| Headache | 61.8 (42/68) | 69.8  (60/86) | 71.3 (241/338) | 66.9  (83/124) | 57.4  (310/540) | 56.9  (422/742) |
| Myalgia | 76.5 (52/68) | 66.3  (57/86) | 82.2 (278/338) | 71.8  (89/124) | 58.7  (317/540) | 60.4  (448/742) |
| Arthralgia | 68.9 (31/45) | 51.1  (24/47) | 77.0 (107/139) | 65.3  (81/124) | 55.9  (195/349) | 59.9  (223/372) |
| Malaise | 95.6 (65/68) | 93.0  (80/86) | 91.4 (308/337) | 88.7  (110/124) | 84.8  (458/540) | 85.5  (635/743) |
| Decreased appetite | 57.8 (26/45) | 48.9  (23/47) | 63.3 (88/139) | 57.3  (71/124) | 35.8  (125/349) | 38.7  (144/372) |
| Nausea | 24.4 (11/45) | 23.4  (11/47) | 32.4 (45/139) | 26.6  (33/124) | 14.3  (50/349) | 19.6  (73/372) |
| Diarrhea | 6.7  (3/45) | 19.1  (9/47) | 18.7 (26/139) | 12.1  (15/124) | 6.0  (21/349) | 14.2  (53/372) |
| Cough | 95.6  (65/68) | 100 (86/86) | 96.2 (325/338) | 93.5  (116/124) | 93.5  (505/540) | 84.6  (626/740) |
| Cough with sputum | 77.8 (35/45) | 83.0 (39/47) | 56.8 (79/139) | 43.5  (54/124) | 59.0  (206/349) | 51.9  (193/372) |
| Dyspnea | 23.5 (16/68) | 25.6 (22/86) | 16.9 (57/338) | 3.2  (4/124) | 19.5  (105/539) | 16.4  (122/742) |
| Tachypnea | 2.2  (1/45) | 2.1  (1/47) | 3.6  (5/139) | 1.6  (2/124) | 0.9  (3/349) | 3.8  (14/372) |
| Rhonchi | 46.7 (21/45) | 42.6 (20/47) | 23.0 (32/139) | 6.5  (8/124) | 23.8  (83/349) | 23.1  (86/372) |
| Wheezing | 28.9 (13/45) | 27.7 (13/47) | 12.2 (17/139) | 3.2  (4/124) | 13.8  (48/349) | 12.4  (46/372) |
| Need for O_2_ | 2.2  (1/45) | 0  (0/47) | 0  (0/139) | 0  (0/124) | 1.4  (5/349) | 1.6  (6/372) |
| Decreased SaO_2_ | 2.2  (1/45) | 6.4  (3/47) | 2.9  (4/136) | 1.7  (2/118) | 5.9  (20/341) | 5.2  (19/368) |
| Sore throat | 44.1 (30/68) | 64.0 (55/86) | 64.3 (216/336) | 66.1  (82/124) | 70.1  (377/538) | 70.5  (524/743) |
| Coryza | 68.9 (31/45) | 87.2 (41/47) | 74.8 (104/139) | 85.5  (106/124) | 89.1  (311/349) | 78.5  (292/372) |
| Altered smell | 15.6 (7/45) | 14.9  (7/47) | 12.9 (18/139) | 17.7  (22/124) | 18.9  (66/349) | 12.6  (47/372) |
| Altered taste | 20.0 (9/45) | 12.8  (6/47) | 13.7 (19/139) | 16.9  (21/124) | 17.2  (60/349) | 11.8  (44/372) |

^a^ Positive for adenovirus, human rhinovirus, enterovirus, bocaviruses, parainfluenza viruses or seasonal coronaviruses.

^b^ Negative for all viruses and bacteria tested.

hMPV = human metapneumovirus; RSV = respiratory syncytial virus.

**TABLE S4** Association between in-hospital mortality among hospitalized adults who tested positive for human metapneumovirus (*n* = 232).

| **Independent variable** | **In-hospital death, % (n/Total)** | | **Odds ratio (95% CI)** | | |
| --- | --- | --- | --- | --- | --- |
|  | **Yes** | **No** | **Univariable models** | **Multivariable model 1** | **Multivariable model 2** |
| Sex (female = 1) | 52.9 (9/17) | 53.5 (115/215) | 0.97 (0.37–2.61) | – | – |
| Age (continuous, 1-year increase) | 80.1 (11.2) ^a^ | 75.7 (13.5) ^a^ | 1.03 (0.99–1.07) | – | 1.02 (0.98–1.07) |
| LTCF resident | 35.3 (6/17) | 7.4 (16/215) | 6.83 (2.21–19.98) | 8.73 (2.63–29.15) | 7.74 (2.30–26.16) |
| Cardiovascular disease | 76.5 (13/17) | 69.8 (150/215) | 1.31 (0.46–4.43) | – | – |
| Respiratory disease | 41.2 (7/17) | 27.4 (59/215) | 1.88 (0.68–4.99) | 2.29 (0.76–6.76) | 2.36 (0.78–7.04) |
| Diabetes | 23.5 (4/17) | 17.2 (37/215) | 1.59 (0.46–4.59) | – | – |
| Hepatic disease | 0 (0/17) | 6.0 (13/215) | – | – | – |
| Renal disease | 5.9 (1/17) | 15.8 (34/215) | 0.48 (0.05–2.01) | – | – |
| Neurodegenerative disease | 17.6 (3/17) | 7.4 (16/215) | 2.92 (0.70–9.59) | – | – |
| Cancer | 47.1 (8/17) | 24.2 (52/215) | 2.79 (1.03–7.45) | 4.51 (1.50–14.35) | 4.51 (1.50–14.47) |
| Immunosuppression | 5.9 (1/17) | 5.6 (12/215) | 1.48 (0.15–678) | – | – |
| Viral co-detections | 11.8 (2/17) | 7.0 (15/215) | 2.09 (0.39–7.64) | – | – |
| Bacterial co-detections | 16.7 (2/12) | 16.4 (30/183) | 1.20 (0.22–4.41) | – | – |

^a^ Reported as mean (standard deviation)

CI = confidence interval; LTCF = long-term care facility.
